# E1A expression dysregulates IL-8 production and suppresses IL-6 production by lung epithelial cells

**DOI:** 10.1186/1465-9921-6-111

**Published:** 2005-09-26

**Authors:** Arjen van den Berg, Mieke Snoek, Henk M Jansen, René Lutter

**Affiliations:** 1 Department of Pulmonology, Academic Medical Center, University of Amsterdam, Amsterdam, The Netherlands; 2 Department of Experimental Immunology, Academic Medical Center, University of Amsterdam, Amsterdam, The Netherlands

## Abstract

**Background:**

The adenoviral protein E1A has been proposed to play a role in the pathophysiology of COPD, in particular by increasing IL-8 gene transcription of lung epithelial cells in response to cigarette smoke-constituents such as LPS. As IL-8 production is also under tight post-transcriptional control, we planned to study whether E1A affected IL-8 production post-transcriptionally. The production of IL-6 by E1A-positive cells had not been addressed and was studied in parallel. Based on our previous work into the regulation of IL-8 and IL-6 production in airway epithelial cells, we used the lung epithelial-like cell line NCI-H292 to generate stable transfectants expressing either E1A and/or E1B, which is known to frequently co-integrate with E1A. We analyzed IL-8 and IL-6 production and the underlying regulatory processes in response to LPS and TNF-α.

**Methods:**

Stable transfectants were generated and characterized with immunohistochemistry, western blot and flow cytometry. IL-8 and IL-6 protein production was measured by ELISA. Levels of IL-8 and IL-6 mRNA were measured using specific radiolabeled probes. EMSA was used to assess transcriptional activation of relevant transcription factors. Post-transcriptional regulation of mRNA half-life was measured by Actinomycin D chase experiments.

**Results:**

Most of the sixteen E1A-expressing transfectants showed suppression of IL-6 production, indicative of biologically active E1A. Significant but no uniform effects on IL-8 production, nor on transcriptional and post-transcriptional regulation of IL-8 production, were observed in the panel of E1A-expressing transfectants. E1B expression exerted similar effects as E1A on IL-8 production.

**Conclusion:**

Our results indicate that integration of adenoviral DNA and expression of E1A and E1B can either increase or decrease IL-8 production. Furthermore, we conclude that expression of E1A suppresses IL-6 production. These findings question the unique role of E1A protein in the pathophysiology of COPD, but do not exclude a role for adenoviral E1A/E1B DNA in modulating inflammatory responses nor in the pathogenesis of COPD.

## Background

Chronic obstructive pulmonary disease (COPD) is characterized by chronic inflammation and irreversible airflow obstruction [1], and is associated with cigarette smoking. Not all smokers, however, develop COPD. Hogg and co-workers have put forward the concept that the presence of the adenoviral E1A DNA and protein in airway structural cells, leading to enhanced IL-8 production in response to endotoxin exposure, may be related to the development of COPD. First they showed by PCR analysis that lung tissue from COPD patients contained more E1A DNA than lung tissue from matched non-COPD smokers [2]. Subsequently, and in line with the presence of E1A DNA, E1A protein was found to be expressed in airway and alveolar epithelial cells from COPD patients [3]. Based on the model of genomic integration of adenoviral DNA proposed by Graham [4], it is likely that a second early adenoviral gene, E1B, is also integrated and possibly expressed, but this was not investigated. As E1A interacts with a large number of regulatory proteins and as epithelial cells express inflammatory proteins, it was postulated that E1A protein modifies the expression of these inflammatory proteins. Stable E1A transfectants of alveolar type-II-like A549 cells indeed showed increased IL-8 [5] and ICAM-1 [6] expression specifically in response to LPS, which is a major constituent of cigarette smoke. Similar results were obtained with E1A-transformed bronchial epithelial cells transfected with both the E1A and E1B gene [7]. The *in vivo *relevance of E1A expression was illustrated with a guinea pig model, showing an enhanced inflammatory response to cigarette smoke in animals with lung tissue containing E1A DNA [8].

The increased *in vitro *production of IL-8 and ICAM-1 by E1A transfectants to LPS appeared to be dependent on an enhanced transcriptional activity, involving activation of NFκB [5,7]. Our previous studies into the regulation of IL-8 and IL-6 production by lung epithelial-like NCI-H292 cells indicated that, besides transcriptional regulation through NFκB, AP-1 and C/EBP activation, post-transcriptional regulation, exemplified by a modified mRNA degradation, was a major means of regulating IL-8 and IL-6 responses [9-11]. Similar findings were obtained with another lung epithelial cell line, Calu-3 cells, as well as primary bronchial epithelial cells. In fact, lung epithelial cells with a decreased rate of mRNA degradation displayed hyperresponsive IL-8 and IL-6 production, similar to that observed for E1A-expressing A549 cells [5]. Therefore, we hypothesized that, expression of E1A in lung epithelial-like cells may lead to stabilization of IL-8 mRNA paralleled by increased IL-8 production in response to LPS and to TNF-α. In parallel, we analyzed IL-6 production, which was anticipated to be decreased, as E1A is known to inhibit IL-6 transcription [13].

To investigate this, we generated stable transfectants of NCI-H292 cells expressing E1A. As the E1B gene is frequently co-integrated with that of E1A, and as E1B protein modifies E1A functions, we also generated stable E1A- and E1B-transfectants. Stable transfectants of E1B and of the empty vector expressing green fluorescent protein (GFP)-tagged zeocin-resistance protein served as controls.

## Materials and methods

### Constructs

In order to construct pTracerSV40-ZeocinGFP vectors (Invitrogen, Paisley, UK) expressing E1A, E1B, or both proteins, pAt153-Xho [14] (a kind gift from Dr. Robert Vries, LUMC, Department of Molecular Cell Biology, Leiden, The Netherlands), containing the first 5789 bp of the Ad5 genome was used as donor construct. To construct the vector pTracer-E1A, containing E1A only, pAt153-xho was digested with Sst1 and the 7K fragment, containing E1A and vector sequence, was isolated and ends were blunted with T4 DNA polymerase. Subsequently this fragment was digested with EcoR1 and the 1774 bp fragment was isolated and ligated into EcoR1/EcoRV digested pTracer-SV40.

To construct the pTracer-E1B, containing only E1B (both 19K- and 55K-E1B proteins), pAt153-Xho was digested with HPA1 and APA1 and the isolated fragment was ligated into pTracer-SV40. The vector pTtracer-E1AB was constructed by digestion of pAt153-Xho with EcoR1 and APA1 and subsequent ligation.

All constructs were verified by sequencing (BigDye sequencing kit, ABI, Foster City, CA) and restriction analysis.

### Generation of stable clones

Human lung mucoepidermoid carcinoma derived NCI-H292 cells (CRL 1848; American Type Culture Collection (ATCC), Manassas, VA) were grown to 90% confluency in a 75 cm^2 ^culture flask, as described before [11]. Before transfection, growth medium was replaced with 10 ml medium without penicillin and streptomycin. Twenty-five μg of vector DNA was mixed with 60 μl of Lipofectamine 2000 (Invitrogen) in a volume of 3 ml Optimem-1 (Invitrogen) and layered onto NCI-H292 cells resulting in 20–30% GFP-positive cells after 24 h. Stable clones were obtained by selection in medium containing 100 μg/ml Zeocin (Invitrogen), of which 250 μl/well was plated in 48-wells plates at a concentration of 6 cells/ml. Medium was replaced twice a week. After formation of colonies, screening of clones was initially performed by immunohistochemistry in 96-well plates. Clones positive for E1A or E1B were selected and expression of E1A or E1B was confirmed by Western blot. Clones negative for E1A or E1B as determined by immunohistochemistry were discontinued. Monoclonality of clones was tested by flowcytometry analyzing both GFP and E1A.

### Cell culture

Clones were cultured and propagated as described before for NCI-H292 cells [11], with the exception that 100 μg/ml Zeocin was added to the medium. Before experiments, cells were cultured one week without Zeocin, and all experiments were performed in Zeocin-free medium.

For cytokine release, 6 × 10^5 ^cells were plated and grown overnight in 500 μl in 24-well plates. For isolation of mRNA and nuclear extracts, 30 × 10^5 ^cells were plated and grown overnight in 2.5 ml in 6-well plates.

### Immunohistochemistry

Cells were fixed with 4% (v/v) paraformaldehyde/0.1% (w/v) Saponin and subsequently incubated with ice-cold methanol for 1 min. To prevent non-specific signals, cells were incubated for 1 h with 5% (w/v) BSA containing 0.1% (w/v) sodiumazide and 0.1% (v/v) H_2_O_2 _and 0.1% Saponin. Then, the cells were incubated with primary antibody (M73 (Santa Cruz Biotechnologies, Santa Cruz, CA) for E1A, 1G11 for E1B-19K or 9C10 E1B-55K [15], both kind gifts from Dr. Robert Vries (LUMC, Leiden, Netherlands) diluted 1:500 in PBS/0.5%BSA/0.1% Saponin (PBSAP) overnight at 4°C. Next, cells were washed 3× with PBS/0.1% Saponin and incubated with 1:250 biotinylated goat-anti-mouse-IgG in PBSAP (DakoCytomation Glostrup, Denmark) for 1 h followed by 3 wash steps. Finally, cells were incubated with Streptavidin-Horseradish Peroxidase (HRP, DakoCytomation) (1:250 in PBSAP) for 30 min, washed and developed with AEC staining solution (Vector Laboratories, Burlingame, CA).

### E1A FACS

Cells were trypsinized and fixed with 4% paraformaldehyde/0.1% Saponin. To prevent aspecific binding, cells were incubated for 30 min with PBS/5% BSA/0.1% Saponin. Then, cells were incubated with the primary antibody M73 (1:500 in PBSAP) for 30 min and subsequently with biotinylated goat anti-mouse IgG (1:250 in PBSAP) for 30 min. Next, cells were labeled with Streptavidin-Allophycocyanin (DakoCytomation, 1:100 in PBSAP) and analyzed by a FACSCalibur flow cytometer and CellQuest Pro software (BD Biosciences). All incubations were performed at RT on a shaking platform. Between incubations, cells were washed twice with PBSAP.

### Western blot

Lysates were prepared by scraping cells (± 5 × 10^6^) in lysis buffer (1% (w/v) NP40, 10 mM Tris-HCl pH 7.4, 150 mM NaCl, 5 mM EDTA, 1 mM phenylmethylsulphonyl-fluoride (PMSF)). Lysates were cleared by centrifugation at 13,000 g for 15 min. Protein contents in cell lysates were determined using Coomassie Plus protein assay reagent (Pierce, Rockford, IL, USA). Fifty μg of protein/lane was separated by SDS-PAGE under reducing conditions. After transfer to nitrocellulose (Hybond-C, Amersham, Buckinghamshire, UK), blots were blocked with 5% (w/v) non-fat dry milk in TBST (10 mM Tris, 150 mM NaCl and 0.05% (v/v) Tween-20, pH 8.0), and were probed overnight at 4°C with M73 antibody 1:1000 diluted in TBST containing 2.5% non-fat dry milk. Immunoreactive proteins were visualized using HRP-conjugated Ig (Goat-anti-mouse for M73 and 9C10 or Goat-anti-Rat for 1G11) and enhanced chemiluminescence (ECL, Amersham).

### Determination of IL-6 and IL-8 protein

Cells were exposed for 8 h to various doses of TNF-α (rhTNF-α, R&D Systems, Minneapolis, MN, USA) or LPS derived from *E.Coli *K-235 (L2018, Sigma-Aldrich, St. Louis, MO) up to 5 ng/ml and 1 μg, respectively. The amount of IL-6 and IL-8 in culture supernatants was measured by sandwich ELISA, as described before [9,12].

### mRNA half-life analysis

Cells were stimulated with TNF-α (5 ng/ml) or LPS (0.1 μg/ml) for one hour before 5 μg/ml actinomycin D (Sigma-Aldrich) was added to block further transcription. Total RNA was extracted with TriZol (Invitrogen) at 0, 40 and 80 minutes after Actinomycin D addition. The amount of IL-6, IL-8 and GAPDH mRNA was determined by dotblotting and hybridization with specific ^32^P-labeled probes for IL-6, IL-8 and GAPDH, which have been extensively validated for specificity in our samples by Northern blot as described [10,11]. Blots were quantified using a phosphorimager and variable loading was corrected for by expressing mRNA levels relative to that of the housekeeping gene GAPDH. mRNA half-life was calculated using linear regression.

### Isolation of nuclear extracts and electrophoretic mobility shift assay (EMSA)

Nuclear extracts were isolated after 1 h stimulation with 5 ng/ml TNF-α and 0.1 μg LPS as described [10,11]. Protein concentrations were measured as described above. Five μg of the nuclear extracts were incubated with ^32^P-labeled oligonucleotides at 4°C for 1 h and separated on a 4% non-reducing poly-acrylamide gel at slowly increasing voltages (60–220 V). Bands were identified by supershift using 1 μg of antibodies against p65 for NF-κB, c-fos and c-jun for AP-1, and C/EBP-β for C/EBP (Santa Cruz Biotechnology Inc., Santa Cruz, CA) and by competition with cold probe. The intensity of the bands was quantified using a phosphorimager. The following oligonucleotides were used in the EMSA:

NF-κB, 5'-TTGCAAATCGTGGAATTTCCTCTGACATAA-3';

AP-1, 5'-TTAAGTGTGATGACTCAGGTTTAA-3';

C/EBP, 5'-TTAAAGGACGTCACATTGCACAATCTTAATAA-3'.

### Construction of siRNA

siRNAs directed against both 12S and 13S E1A mRNA were designed using the Ambion siRNA target finder  using accession code AY147066.

The following sequences were selected:

AACTGTATGATTTAGACGTGA (Start position in sequence: 134)

AAGTGAAAATTATGGGCAGTG (Start position in sequence: 599)

AATGCAATAGTAGTACGGATA (Start position in sequence: 671)

AATTTTTACAGTTTTGTGGTT (Start position in sequence: 660)

AATGTATCGAGGACTTGCTTA (Start position in sequence: 800)

AAGATCCCAACGAGGAGGCGG (Start position in sequence: 723, published in [16])

Oligonucleotides were then designed using the Ambion siRNA Template Design Tool . siRNAs were constructed with the Silencer™ siRNA Construction Kit (Ambion, Austin, TX) according to the manufacturer's instructions. As a control for proper synthesis and transfection efficiency, the GAPDH siRNA template included in the kit was used. One to 10 pmol of siRNA was mixed with 0.5 μl Lipofectamine 2000 (Invitrogen) in 50 μl Optimem-1 medium (Invitrogen) according to the manufacturer's instructions and transferred to a well of a 48-wells plate with cells at 30–50% confluency. Assessment of gene silencing was performed 24- or 48 post-transfection by PCR and immunohistochemistry.

### E1A and GAPDH PCR

Single strand cDNA was synthesized from total RNA isolated with Trizol (Invitrogen) using 500 ng of oligo(dT)_15 _and 100 units of Superscript II (Gibco/Brl) in a 50 μl volume. One μl of cDNA was used in the PCR reaction (50 mM KCl, 2 mM MgCl_2_, 10 mM Tris-HCl (pH 9.0) 200 mM of each dNTP, 0.1% Triton X-100, 200 nM of each primer and 1.25 U of Taq DNA polymerase (Promega, Madison, WI)). For E1A, the PCR conditions were 30 thermal cycles at 94°C for 1 min, 59°C for 1 min and 72°C for 1 min, followed by a final extension at 72°C for 10 min. The following primers were designed using primer 3  and accession code X02996:

5'-GTGACGACGAGGATGAAGA-3' (bp 395–413);

5'-ACGGCAACTGGTTTAATGG-3' (bp 614–632).

This primer set produces a 238 bp product for 12S E1A mRNA and a 375 bp product for 13S E1A mRNA. Unspliced E1A mRNA yields a 495 bp product.

For GAPDH, the PCR conditions were 25 thermal cycles at 94°C for 1 min, 55°C for 1 min and 72°C for 1 min, followed by a final extension at 72°C for 10 min. The following primers were designed using primer 3 and accession code NM_002046:

5'-ATGAAGGTCGGAGTCAACG-3' (bp 86–103);

5'-TGAAGACGCCAGTGGACTC-3' (bp 364–382):

This primer set produces a 296 bp product.

## Results

### Generation of stable transfectants and expression of target proteins

**Figure 1:**
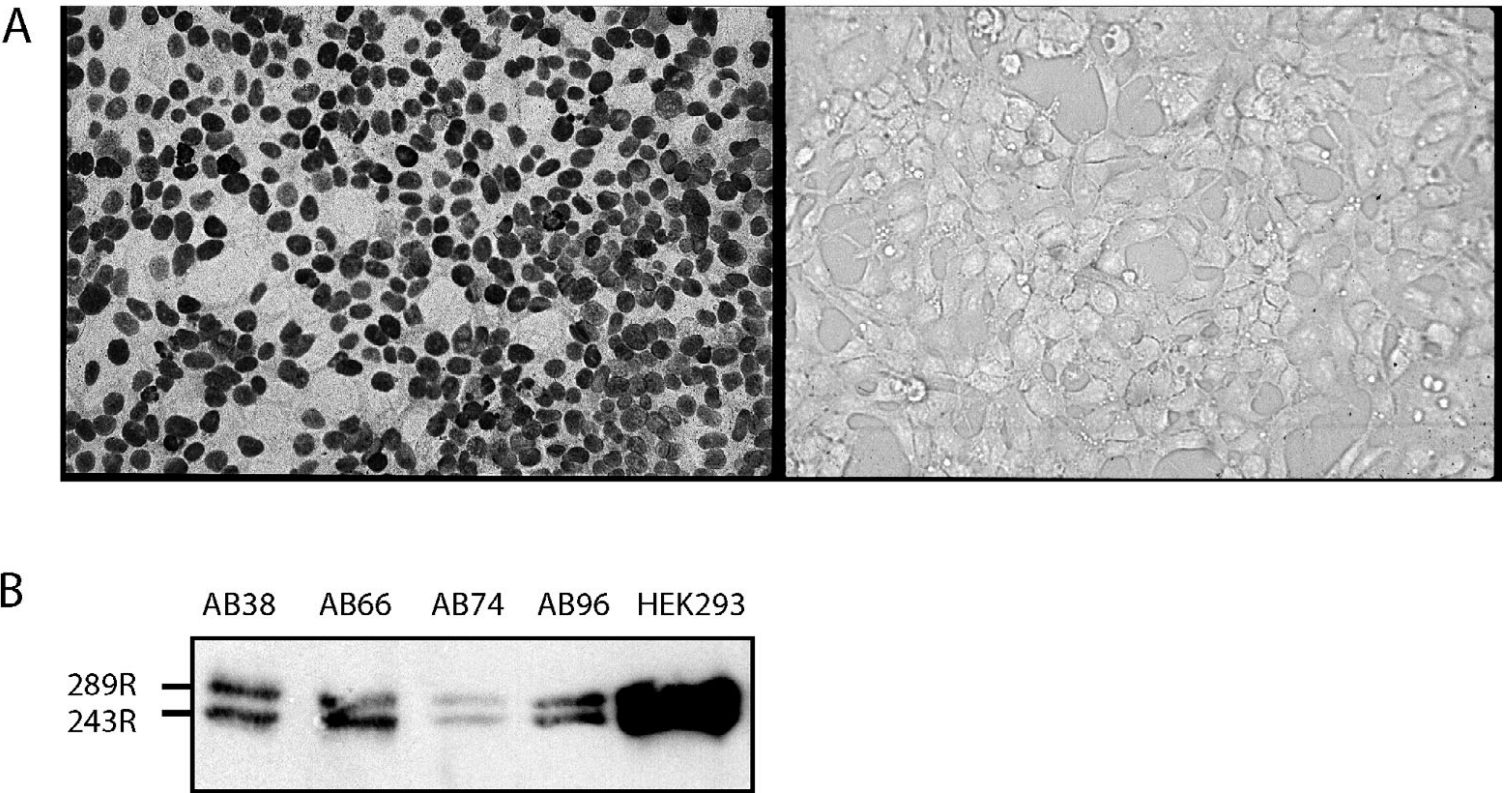
**E1A expression in AB clones**. IHC for clone AB96 and for other AB clones demonstrated a strict nuclear localization of expressed E1A (A, left panel) in E1A-positive clones. The right panel is stained with a control IgG (200 fold magnification). Expression of E1A by AB clones was confirmed by Western blot (B), with HEK293 cells as positive control. Please note the equal expression of both the 289R- and the 243R-E1A proteins.

Constructs of E1A, E1B and E1A plus E1B in the pTracerSV40-Zeo vector were used to generate stable transfectants of NCI-H292 cells. Over 150 zeocin-resistant GFP-positive clones transfected with the E1A construct were generated, but none of these clones expressed E1A at a level detectable by immunohistochemistry (IHC). As expression of E1A leads to apoptosis in non-ras-transformed cells [17,18], it is likely that NCI-H292 cells expressing E1A protein underwent apoptosis. Transfection with E1A plus E1B yielded 144 zeocin-resistant clones, 6 of which (designated AB-clones) were found to express E1A by IHC (Fig 1A). Western blot showed that both E1A gene products, the 289R and 243R E1A, were expressed at equal levels (Fig 1B). The E1A-positive clones expressed low levels of either 19K or 55K E1B by Western blot which was not detectable by IHC. Transfection with the E1B construct yielded 30 zeocin-resistant clones, 6 of which (designated B-clones) were positive for the 55K E1B protein as determined by IHC. As controls, we generated 7 stable transfectants with the empty vector expressing GFP (designated T clones).

### Effect of E1A expression on IL-8 and IL-6 secretion in response to LPS and TNF-α

We next evaluated the TNF-α- and LPS-induced IL-8 and IL-6 responses of the various clones. Hyperresponsive and hyporesponsive clones are defined respectively as clones with a significant (p < 0.05) 2.5-fold increased or 2.5-fold decreased (i.e. 0.4 times) IL-8 or IL-6 production relative to the mean of the control (T) clones, for all tested doses of a stimulus. Normoresponsive clones are those that stay within this 2.5-fold range. This definition is based on the observation of Hogg and coworkers, who showed at least a 2.5-fold increase in IL-8 production in response to 0.01 μg/ml LPS for an E1A-positive clone compared to an E1A-negative clone [5].

**Figure 2:**
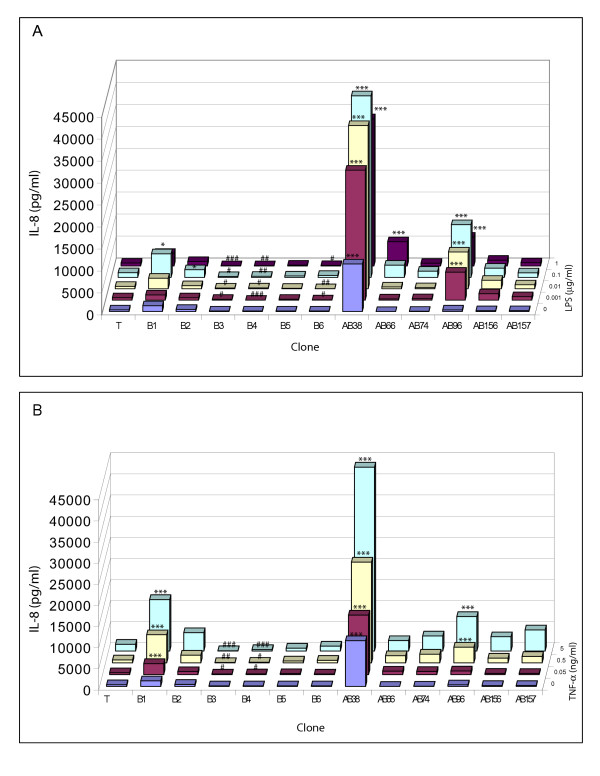
**IL-8 production by various clones in response to exposure to LPS and TNF-α**. Equal cell numbers from clones were exposed to a concentration range of LPS (see z-axis; 0–1 μg/ml, A) or TNF-α (0–5 ng/ml, B) for 8 hrs. IL-8 in culture supernatant was measured by ELISA. T represents the mean IL-8 production (in pg/ml; y-axis) of 7 T-clones. Individual B and AB clones as designated on the x-axis. Data are shown as the mean of two independent experiments (triplicate samples). Due to the representation as a 3D-matrix, no standard deviation can be shown. Asterisks indicates significant (* = p < 0.05, ** = p < 0.01, *** = p < 0.001) hyperresponsiveness, # indicates significant hyporesponsiveness (# = p < 0.05, ## = p < 0.01, ### = p < 0.001).

**Figure 3:**
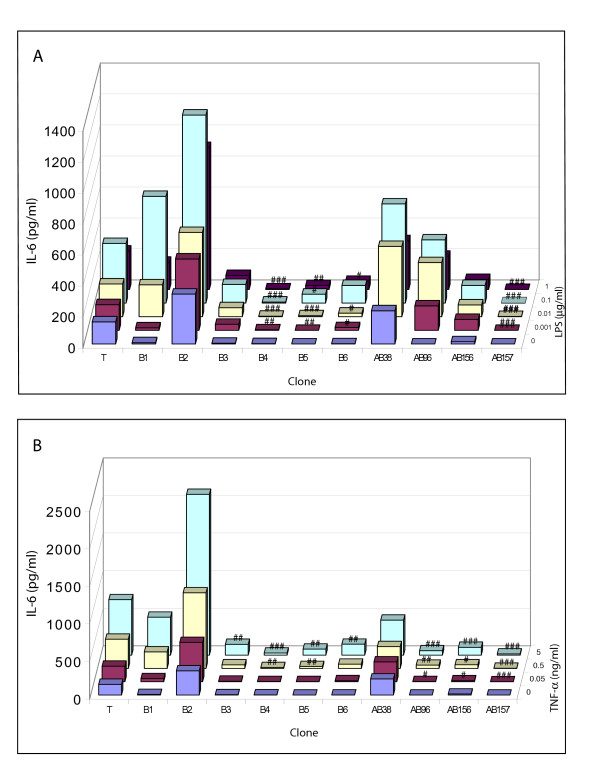
**IL-6 production in response to exposure to LPS and TNF-α**. Equal cell numbers from clones were exposed to a concentration range of LPS (see z-axis; 0–1 μg/ml, A) or TNF-α (0–5 ng/ml, B) for 8 hrs. IL-6 in culture supernatant was measured by ELISA. T represents the mean IL-6 production (in pg/ml; y-axis) of 7 T-clones. Individual B and AB clones as designated on the x-axis. Statistics as described in figure 2.

Of the 6 AB clones tested, 2 clones (AB38 and AB96) showed a hyperresponsive IL-8 production in response to both LPS and TNF-α, whereas the other 4 did not (Fig 2, Table 1). One B-clone (B1) was hyperresponsive to TNF-α but not to LPS, whereas two B-clones (B3 and B4) were hyporesponsive to both TNF-α and LPS. With respect to IL-6, none of the tested clones displayed a hyperresponsive IL-6 production in response to LPS or TNF-α (Fig 3, Table 1). In fact, one AB clone (AB157), and two B clones (B4 and B5) displayed hyporesponsive IL-6 production to LPS. All AB clones except AB38 showed hyporesponsive IL-6 production to TNF-α (Fig 3B, Table 1), and the same two B clones (B4 and B5) that had a hyporesponsive IL-6 production to LPS also displayed a hyporesponsive IL-6 production to TNF-α.

**Table 1:** Number of B- and AB-clones displaying hyper- or hyporesponsive IL-8 and IL-6 production to LPS or TNF-α. Clones transfected with E1A plus E1B are designated AB and clones transfected with E1B are designated B. Hyperresponsive and hyporesponsive clones are defined respectively as clones with a significant (p < 0.05) 2.5-fold increased or 2.5-fold decreased (i.e. 0.4 times) IL-8 or IL-6 production relative to the mean of the control (T) clones, for all tested doses of a stimulus.

		**Hyperresponsive**		**Hyporesponsive**		
		
		LPS	TNF-α	LPS	TNF-α	Total
**IL-8**	B	0	1	2	2	6
	AB	2	2	0	0	6
**IL-6**	B	0	0	2	2	6
	AB	0	0	1	3	4

### Analysis of transcriptional activation by EMSA

**Figure 4:**
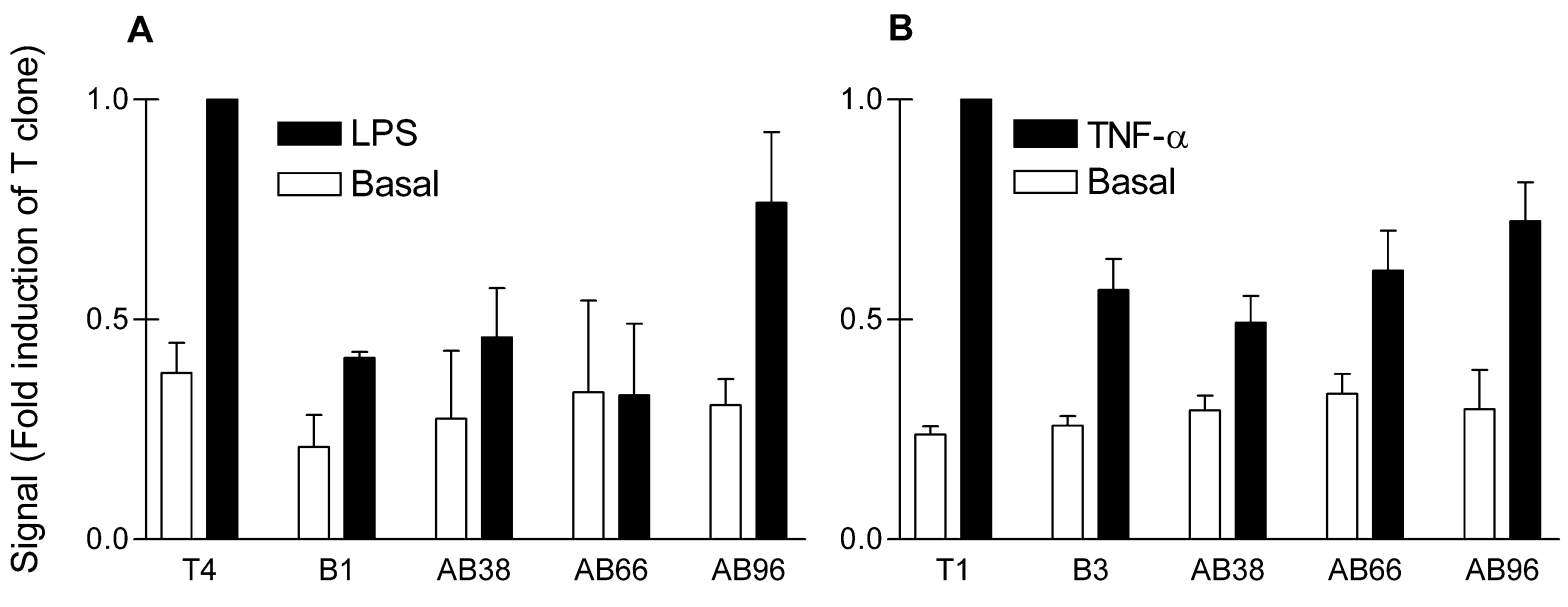
**Analysis of nuclear NFκB recruitment by EMSA**. Equal cell numbers from clones, indicated on the x-axis, were stimulated with 0.1 μg/ml LPS (A) or 5 ng/ml TNF-α (B) for one hour, before nuclear extracts were prepared. Specific bands were identified by supershift using a p65 antibody for NFκB and cold oligo competition. Bands were quantified by phosphorimager and expressed relative to that of the stimulated T clone, which was set at one. Data represent mean ± SEM from 2 independent experiments.

Our previous studies showed that TNF-α- and LPS-induced transcription of IL-8 in NCI-H292 cells is dependent mainly on the transcription factor NFκB and to a lesser extent on AP-1 [11]. We compared activation of NFκB in both AB clones with a hyperresponsive IL-8 production to LPS (AB96 and AB38) to that of a normoresponsive AB-clone (AB66), a T-clone (T4) and a B-clone (B3) (Fig. 4A). We found no further upregulation of NFκB activation by E1A expression. Similar findings were observed upon stimulation with TNF-α. (Fig 4B).

We also determined nuclear recruitment of transcription factors AP-1 and C/EBP; the latter is involved in regulation of IL-6 gene transcription in NCI-H292 cells [10]. Again, we found no altered upregulation of AP-1 or C/EBP recruitment in E1A-expressing clones to either TNF-α or LPS (data not shown).

### Analysis of IL-8 and IL-6 mRNA half-life

**Figure 5:**
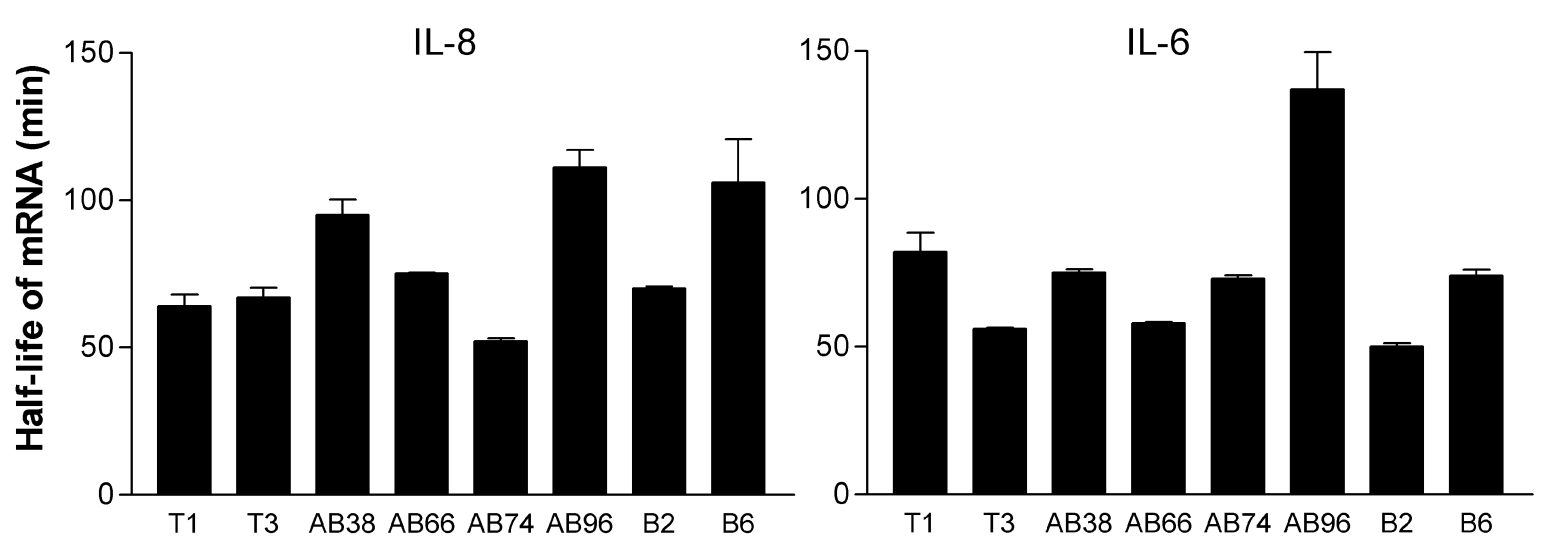
**IL-8 and IL-6 mRNA half-life after exposure to TNF-α**. Equal cell numbers from clones, indicated on the x-axis, were stimulated with 5 ng/ml TNF-α for one hour before 5 μg/ml Actinomycin D (ActD) was added to block further transcription. At 0, 40 and 80 min after ActD addition, total RNA was extracted with TriZol. RNA was dot blotted and hybridized with ^32^P-labelled IL-8, IL-6 and GAPDH probes. Signals were quantified on a phosphorimager and IL-8 and IL-6 mRNA levels were normalized for variable loading using GAPDH mRNA levels. Half-life of the mRNA in the clones was calculated using linear regression. Data represent the mean ± SEM from 2 independent experiments (triplicate samples).

An increased half-life of IL-8 and IL-6 mRNA can be accompanied by an enhanced production of these cytokines [9,12]. Therefore, we tested whether the difference in responsiveness to TNF-α and LPS of the clones was paralleled by alterations in the half-life of IL-8- and IL-6 mRNA. One hyperresponsive AB clone (AB96) tended to have an increased half-life of IL-8 mRNA compared to the normoresponsive T clones (p= 0.06). However, the normoresponsive clone B6 had a similarly increased IL-8 mRNA half-life (Fig. 5A). Moreover, clone AB96, that displayed hyporesponsive IL-6 production, had a relative stable IL-6 mRNA (Fig. 5B). Similar heterogeneous results emerged when clones were stimulated with LPS (data not shown), and thus differences in responsiveness between the various clones did not parallel changes in the half-life of IL-8 and IL-6 mRNA.

### Responses of E1A- and E1B-expressing NCI-H292 subclones

Together, these data indicated that expression of biologically active E1A did not correlate with an enhanced IL-8 production, nor did E1A expression uniformly affect mechanisms regulating IL-8 production. For IL-6, there was a similar heterogeneity, but E1A expression appeared to inversely correlate with IL-6 production. To exclude that the observed heterogeneity in responses was due solely to an intrinsic heterogeneity of the mother cell line, we subcloned NCI-H292 cells and tested IL-6 responses to TNF-α of 15 subclones. We found up to a 15-fold difference in maximal IL-6 responses between clones (this response range is calculated by dividing the maximal response by the minimal response at 5 ng/ml TNF-α for a group of clones; data not shown), which is similar to that for the T-clones (11-fold difference), but less than that for B- and AB-clones, 30- and 40-fold difference, respectively. This suggests that the larger response range in AB- and B-clones is caused by the presence of E1A and E1B DNA, although part of the response range appears due to biological variation of the motherline and/or results from the procedure of subcloning. To further test the latter we cloned an earlier derived subclone and tested TNF-α- and LPS-induced responses from 11 derived sub-subclones. This time, we found a 4-fold difference between clones in their maximal IL-6 and also IL-8 responses, which indicates that there is some variation in the NCI-H292 motherline.

**Figure 6:**
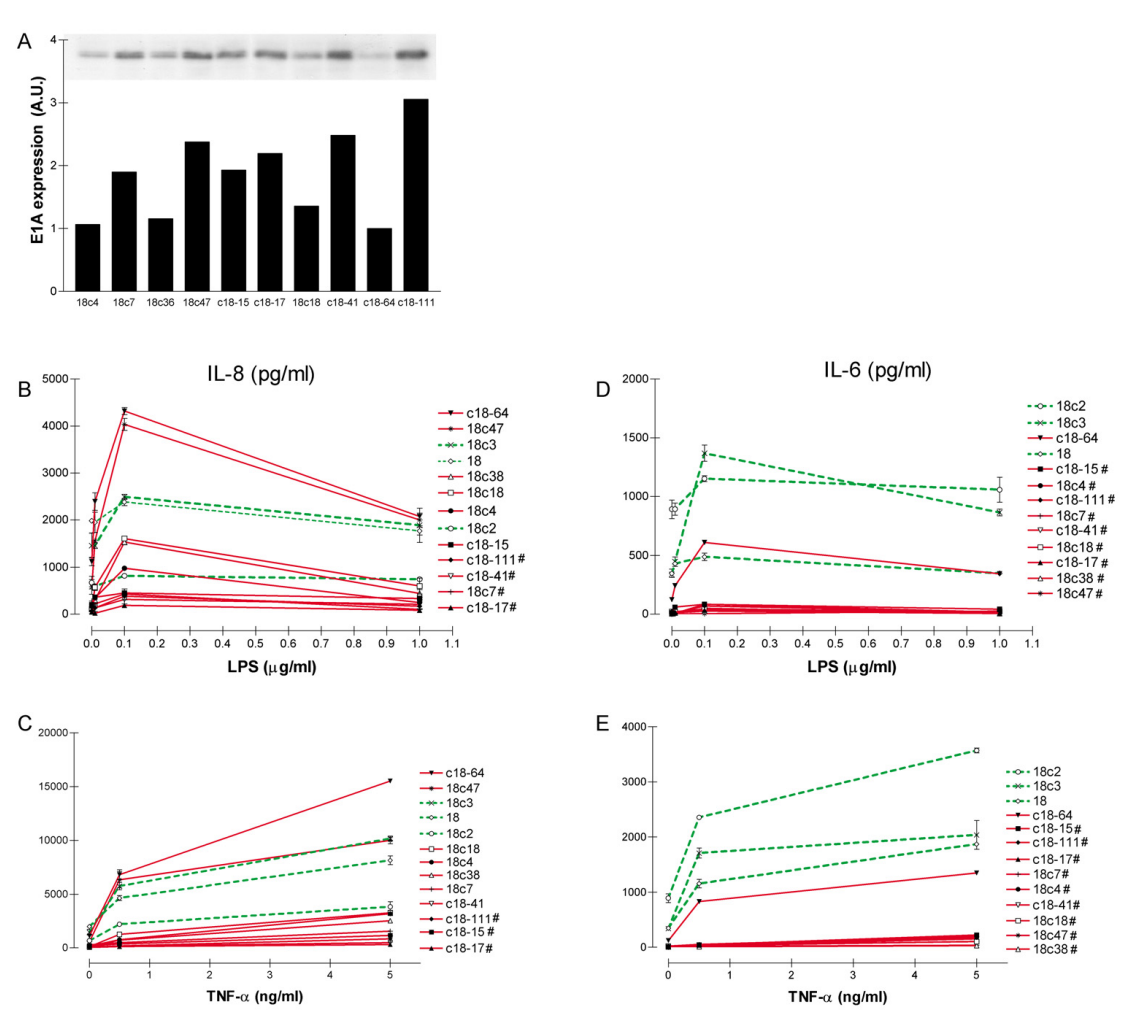
**Expression levels of E1A protein and IL-8 and IL-6 responses of transfected NCI-H292 subclones**. A) Transfection of a NCI-H292 subclone yielded 10 E1A-positive clones. Expression level of E1A in the different clones was determined by Western blot. Exposed films were scanned and quantified. Signal is expressed in arbitrary units. Even though E1A shows as a single band in the printed figure, close examination of the electronic figure reveals both the 289R and the 243R E1A protein. Equal cell numbers from clones were exposed to a concentration series of LPS (0, 0.01, 0.1 and 1 μg/ml, 6B&D) or TNF-α (0, 0.5 and 5 ng/ml, 6C&E) for 8 hrs. IL-8 en IL-6 concentrations in culture supernatant were measured by ELISA. E1A-negative clones are represented by dashed curves, E1A-positive clones by solid curves. For clarity, the clone numbers at the right are ranked for IL-8 and IL-6 production, and hyporesponsive clones (p < 0.05) are marked with #. Data are from one experiment (triplicate samples) out of two independent experiments with similar results.

To confirm the effect of E1A and E1B expression on the response range, we transfected one of the clones derived from subcloning the NCI-H292 motherline, with E1A plus E1B. Ten E1A-positive clones differing in the level of E1A expression were obtained (Fig 6A) and tested for IL-8 and IL-6 responses to LPS and TNF-α (Fig 6B–E). Compared to E1A-negative clones, nine out of ten clones showed a significant hyporesponsive IL-6 production to LPS or TNF-α; the clone with the lowest E1A expression having a normoresponsive IL-6 production. Even though the absolute IL-6 production was much lower in E1A-positive clones, the range of IL-6 production was much larger in the E1A-positive clones than E1A-negative clones, showing difference of upto 130- and 3-fold, respectively.

None of the clones displayed significant hyperresponsive IL-8 production to LPS, whereas 4 out of 10 E1A-positive clones showed hyporesponsive IL-8 production to LPS. Similarly, none of the clones displayed a hyperresponsive IL-8 response to TNF-α, and 3 out of 10 clones showed a hyporesponsive IL-8 production. In line with our previous results, the response range for IL-8 in the E1A-positive cells reached up to 48 for TNF-α and 23 for LPS, whereas the control clones displayed a response range of 3.

### siRNA results

To provide ultimate proof of the role of E1A protein, we attempted to knock down E1A expression using siRNA. Five different siRNA duplexes directed to both 12S and 13S E1A mRNA, coding for the 243R and 289R E1A proteins [19], were generated and transfected into 4 different AB clones. As a positive control, a GAPDH siRNA was used. Transfection of GAPDH siRNA in a final concentration of 4 nM resulted in a specific downregulation of more than 90% of GAPDH mRNA, 24 h and 48 h after transfection, indicating that both siRNA synthesis and transfection protocols were functional. None of the siRNAs directed to E1A downregulated E1A mRNA at a concentration of 4 nM. Transfection of higher concentrations of E1A siRNA (up to 35 nM) resulted in aspecific silencing of both E1A and GAPDH mRNA.

We next used the shRNA DNA vector pSP-E1A (a kind gift from Dr. David Hacker, LBTC EPFL, Lausanne, Switzerland) that was shown to reduce E1A mRNA by 75% in HEK293 cells [16], but due to either a low transfection efficiency of DNA vectors in our clones (<30%) or incompatibility with the U6 promoter, we could not detect downregulation of E1A expression. To circumvent the latter, an siRNA using the same sequence was generated, which neither knocked down E1A mRNA expression in our clones.

## Discussion

The present study aimed to investigate and extend the hypothesis that E1A expression in lung epithelial cells, contributes to increased IL-8 responses. We analyzed a panel of stable transfectants expressing E1A, but we found no uniform effect on IL-8 protein production, whereas the effect on IL-6 production was more uniform. If anything, clones expressing E1A displayed a much larger response range for IL-8 than control clones, suggesting that expression of E1A dysregulates IL-8 production, leading to either an increased or a suppressed IL-8 production. Similar findings were obtained for E1B, indicating that the effect is not specific for E1A.

These findings are in apparent contradiction with those reported by Hogg *et al*., who described an increase in IL-8 production in E1A-expressing ras-transformed alveolar-type II-like A549 cells as well as E1A/E1B-transformed primary bronchial epithelial cells [5,7]. In the present studies we have used lung epithelial NCI-H292 cells, which have been studied extensively in relation to IL-8 and IL-6 production [9-12] and showed regulation similar to that of other lung epithelial cell lines and primary bronchial epithelial cells. For example, effects of IL-17 on TNF-α induced IL-8 and IL-6 secretion were similar and regulated in the same way [20]. Most importantly, post-transcriptional regulation of IL-8 and IL-6 responses, which is a major mode of regulating these responses, are directed by various conditions to a similar extent in H292 cells and primary cells. As NCI-H292 cells are also susceptible to infection by many strains of adenoviruses [21], including serotype 5, we considered NCI-H292 cells a good model to study the effect of E1A on IL-8 and IL-6 production, although we cannot exclude that the apparent contradictory results are due to the use epithelial cells from different origin.

E1A protein expressed in our clones was biologically active as is evident from the reduced IL-6 production by E1A-expressing clones (Fig 6), in line with the reported inhibition of IL-6 gene transcription by the E1A protein [13]. Furthermore, immunohistochemical staining of E1A protein was restricted to the nucleus in all clones, indicative of nuclear recruitment which is essential for E1A protein to display its biological activity. Despite the presence of active E1A protein, and the relation between IL-6 responses and the expression levels of E1A, we found no correlation between the level of E1A protein expression and IL-8 production in the present clones. The E1A gene encodes two primary regulators of viral and cellular gene expression, the E1A-243R and E1A-289R proteins [19]. Distinct biological functions have been described for the 243R and 289R E1A products in mouse lung. Preferential expression of the 243R E1A was associated with cellular hyperplasia and low level of p53-mediated apoptosis, whereas preferential 289R E1A expression led to pro-apoptotic injury and acute pulmonary inflammation [22]. Our clones all expressed the 243R and 289R in an apparent 1:1 ratio as detected by Western blot, however the E1A/E1B transformed primary bronchial epithelial cells as described by Higashimoto *et al*. [7] preferentially expressed the 13S transcript coding for the 289R E1A protein and hence may explain the observed difference. As yet there is no data available on preferential expression of either transcript in lung tissue of COPD patients, or in the A549 *in vitro *model used by Hogg and coworkers. Other reasons for the observed differences may arise simply from the fact that we analyzed a larger panel of clones which allowed us to identify the different effects or from the cooperation of Ras with E1A in A549 cells.

Analysis of the transcriptional and post-transcriptional mechanisms of IL-8 and IL-6 production in the various E1A-expressing clones revealed marked heterogeneity. There was no increased nuclear recruitment of the transcription factors NFκB, AP-1 and C/EBP in E1A-positive cells. Also, there was no change observed in the electrophoretic mobility of the protein-DNA complexes that could indicate a change in the composition of the complex. In fact, clone AB38 showed constitutive expression of high levels of C/EPB activation (data not shown), and had a high basal IL-6 production, but was not hyperresponsive. The normoresponsive clone T4 showed the highest nuclear recruitment of NFκB, but there was no correlation between the recruitment of NFκB and the amount of IL-8 released upon LPS stimulation. With respect to the half-life of IL-8 mRNA, there was a similar heterogeneity. One of the hyperresponsive clones (AB96) had an increased stability of IL-8 mRNA, however, IL-8 mRNA in another hyperresponsive clone (AB38) had a normal half-life. Taken together, there was no uniform effect of E1A expression on the transcriptional and post-transcriptional regulation of IL-8 mRNA. In fact, for some transfectants it is unclear why they produce more IL-8 or less IL-6, as there is no apparent effect on transcription or mRNA degradation. An alternative explanation not addressed in the current study is that translational control may be affected in these transfectants.

Unexpectedly, some E1B transfectants showed similar effects on IL-8 and/or IL-6 production to TNF-α and LPS as E1A transfectants. This may suggest that the mere presence of adenoviral DNA or protein affects the regulation of IL-8 and IL-6 production. A possible explanation is that integration of vector DNA in the genome (E1A and E1B normally co-integrate), a poorly understood process, may affect the expression of a functional gene involved in the cascade controlling IL-8 or IL-6 production, leading to an altered cytokine production. The exact mechanism remains to be determined. An approach could be to generate a panel of inducible transfectants.

The effect of E1A and E1B on the IL-8 production is probably best described by increasing the response range. Subclones of the NCI-H292 mother line showed biological variation in IL-6 and IL-8 production, giving a response range of 15. This variation is comparable to that for NCI-H292 clones transfected with an empty vector, but markedly smaller than that for E1A plus E1B or E1B-transfected clones. Similarly, E1A-expressing clones generated from subclones of NCI-H292 showed an enhanced response range for IL-8, which was at large due to reduced minimal IL-8 production.

Ultimate proof for, or against, a role of the E1A protein in increasing IL-8 production in lung epithelial cells would be to knock down E1A expression using siRNA, allowed by the short half-life (30–120 min) of the E1A protein [23]. We generated 6 different E1A siRNAs, but none reduced E1A mRNA or protein. GAPDH, however, was readily downregulated by our GAPDH siRNA, validating the method we used. The published shRNA vector driven by the RNA polymerase-III U6 promotor, which reduced E1A expression by 75% in HEK293 cells [16], neither reduced E1A expression in our cells. The reason for this failure may be due to incompatibility of the U6 promoter with our cells. Furthermore, steric hindrance by proteins bound to E1A mRNA in NCI-H292 cells may have prevented the siRNA to bind. Hence, it remains elusive whether the dysregulated IL-8 production comes about by the actual viral proteins being expressed, or whether the incorporation of the DNA is sufficient.

The question arises whether there still is a role for E1A expression in the pathogenesis of COPD as proposed by Hogg *et al*. From theirs and an other study [24] it follows that E1A DNA and E1A protein is more abundantly expressed in epithelial cells from COPD patients, though the reason for increased E1A expression in COPD patients remains elusive. Based upon our findings it is doubtful that E1A expression per se contributes to the pathogenesis of COPD by means of an increased IL-8 production. We cannot exclude, however, that E1A DNA integration following adenoviral infection differs from that obtained with our transfection approach. In addition, our transfection approach allowed us to analyze adenoviral integrations that may be very rare events by adenoviral infection. Indeed we found large differences in transfection efficiency for the various constructs. This difference may be explained by the cellular effect of the gene that is expressed. As for transfection with E1A alone, there were no viable clones expressing E1A, which we assume was due to apoptosis. In a later study performed by Hogg *et al *it was also described that with primary bronchial cells, it is not possible to obtain clones expressing only E1A, and that E1B expression is needed together with E1A to generate stable clones. The transfection efficiency was much higher with the non-apoptotic E1B and empty vector. Second, the transfection efficiency may depend on the size of the insert in the vector. The mechanism by which a vector integrates remains largely elusive, but in order to integrate the vector needs to linearize, i.e. break open. The point where the vector breaks is random, and the larger the insert is compared to the rest of the vector, the larger the chance that it breaks in the gene of interest, leading to a reduction in efficiency of inserting the full gene. This may underlie the lower efficiency of the E1A/E1B clones, as this insert is of a similar size as the vector, and 2Kb larger than then E1B alone. The much smaller empty vector containing the Zeocyn resistance GFP tagged protein showed the highest transfection efficiency. Furthermore, incomplete insertion of the E1A/E1B sequence may also lead to expression of E1A without E1B, leading again to cell death. As we observed some clones transfected with E1A and E1B that were positive only for E1B, it is likely that this also occurs.

The previously unrecognized decreased IL-6 production by the E1A- and E1B-expressing clones, could contribute to increased inflammation. IL-6 is regarded a pro-inflammatory mediator, but has also been shown to exert many anti-inflammatory and immunosuppressive effects (see ref. [25] for review). Moreover, in several murine models for pulmonary inflammation IL-6 was shown to protect against lung damage [26-29]. The reduced IL-6 production from epithelial cells thus may play a role in the early pathogenesis of COPD, as it is hypothesized that latent adenoviral infection, accompanied by expression of E1A, is established in early childhood [2]. Even though in exhaled breath condensate of patients diagnosed with COPD high levels of IL-6 are found [30], this IL-6 may be derived from sources other than lung epithelial cells. Notably, the increased IL-6 levels are observed in patients already diagnosed with COPD, and thus do not exclude a role for reduced IL-6 production from epithelial cells in the early pathogenesis of COPD. Whether E1A expression affects the expression of other genes in these transfectants is unknown as yet.

## Conclusion

Taken together, our study does not provide support for an unique role of Ad5 E1A protein in the enhanced IL-8 production. Expression of E1A and E1B, however, did affect the IL-8 response to LPS and TNF-α, as was evident by the increased response ranges, in particular due to a reduced IL-8 production of some clones. Both E1A and E1B reduced IL-6 production, which could play a role during the early pathogenesis of COPD. These results warrant further research into the impact of integrated genomic viral sequences on inflammatory responses.

## Abbreviations

AP-1: Activator Protein-1

C/EBP: CAAT/Enhancer Binding Protein

IL: Interleukin

NFκB: Nuclear Factor κB

TNF: Tumor Necrosis Factor

## Competing interests

The author(s) declare that they have no competing interests.

## Authors' contributions

AB prepared DNA constructs, generated stable transfectants, performed all studies mentioned and drafted the manuscript. MS assisted with ELISAs and carried out dotblot hybridizations. HJ participated in the study design and coordination, and helped to draft the manuscript. RL conceived the study, participated in its design and coordination, and revised the draft.
